# Supplementary figures and images for: Measurement of Volatile Compounds for Real-Time Analysis of Soil Microbial Metabolic Response to Simulated Snowmelt
Source: Front Microbiol. 2021 Jun 23;12:679671. doi: 10.3389/fmicb.2021.679671 (PMC8261151; doi:10.3389/fmicb.2021.679671)

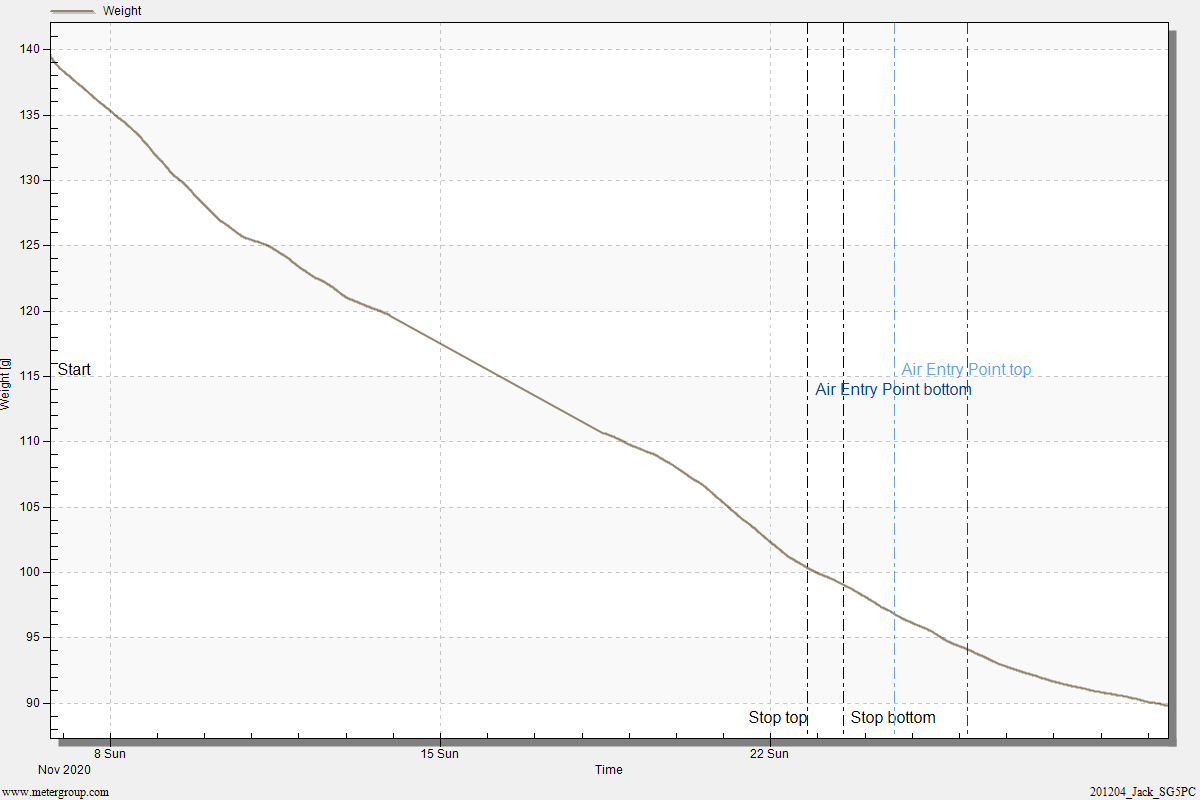

Supplement: Supplementary Data 1 — HYPROP2 and additional data on soil hydraulic properties. [file Data_Sheet_1.ZIP › SupplementaryData1/Soil Weight Over Time.png]

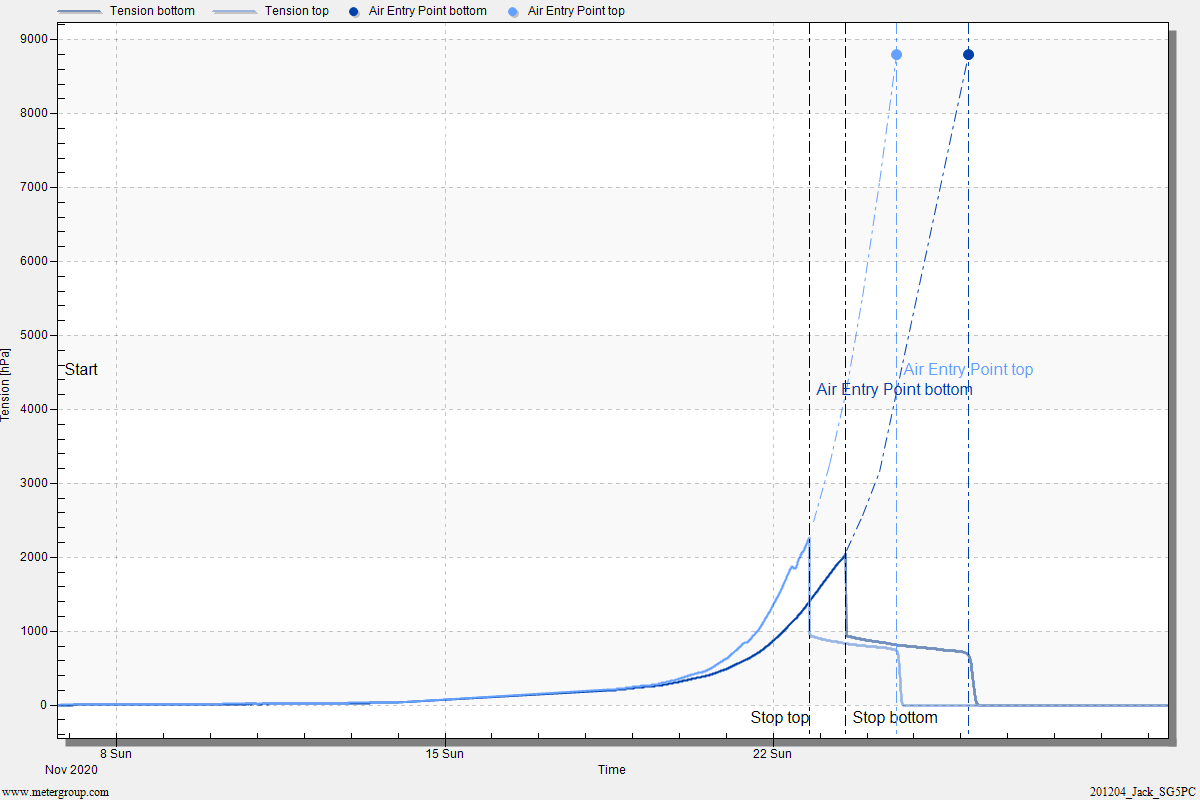

Supplement: Supplementary Data 1 — HYPROP2 and additional data on soil hydraulic properties. [file Data_Sheet_1.ZIP › SupplementaryData1/Tension Over Time.png]

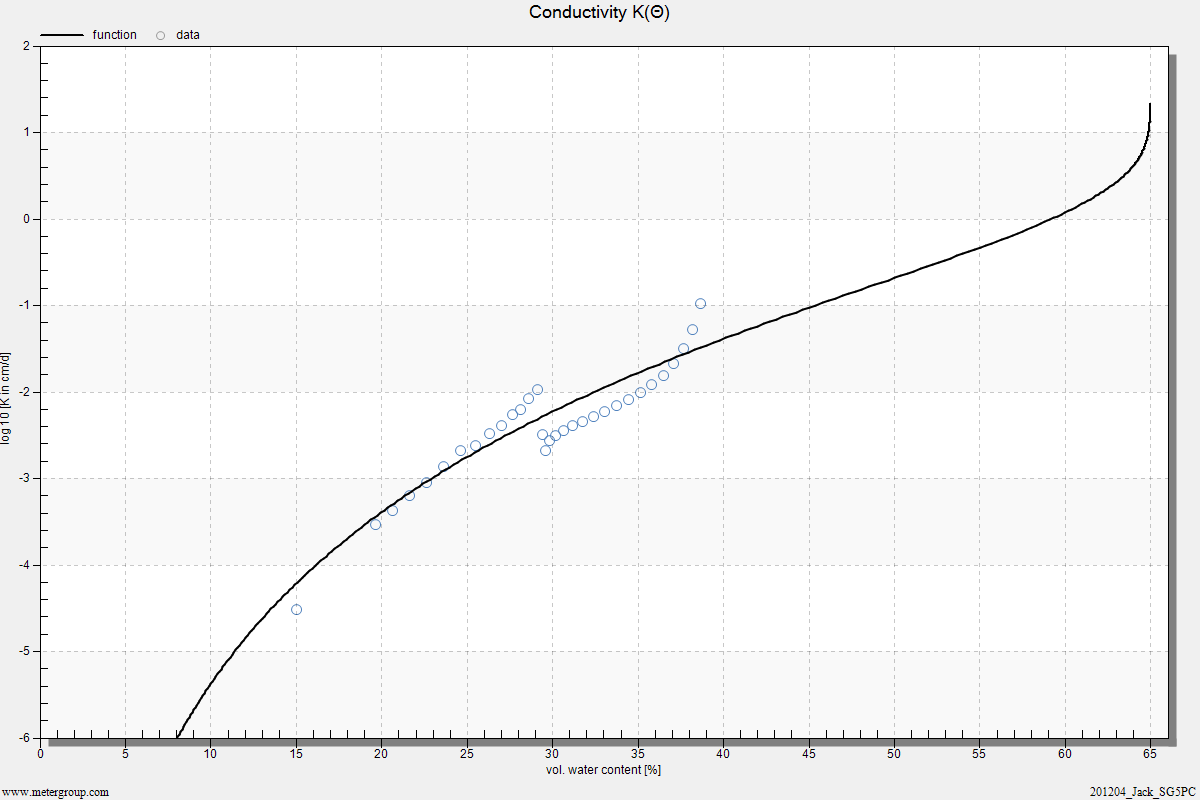

Supplement: Supplementary Data 1 — HYPROP2 and additional data on soil hydraulic properties. [file Data_Sheet_1.ZIP › SupplementaryData1/Soil Conductivity vs Water Content.png]

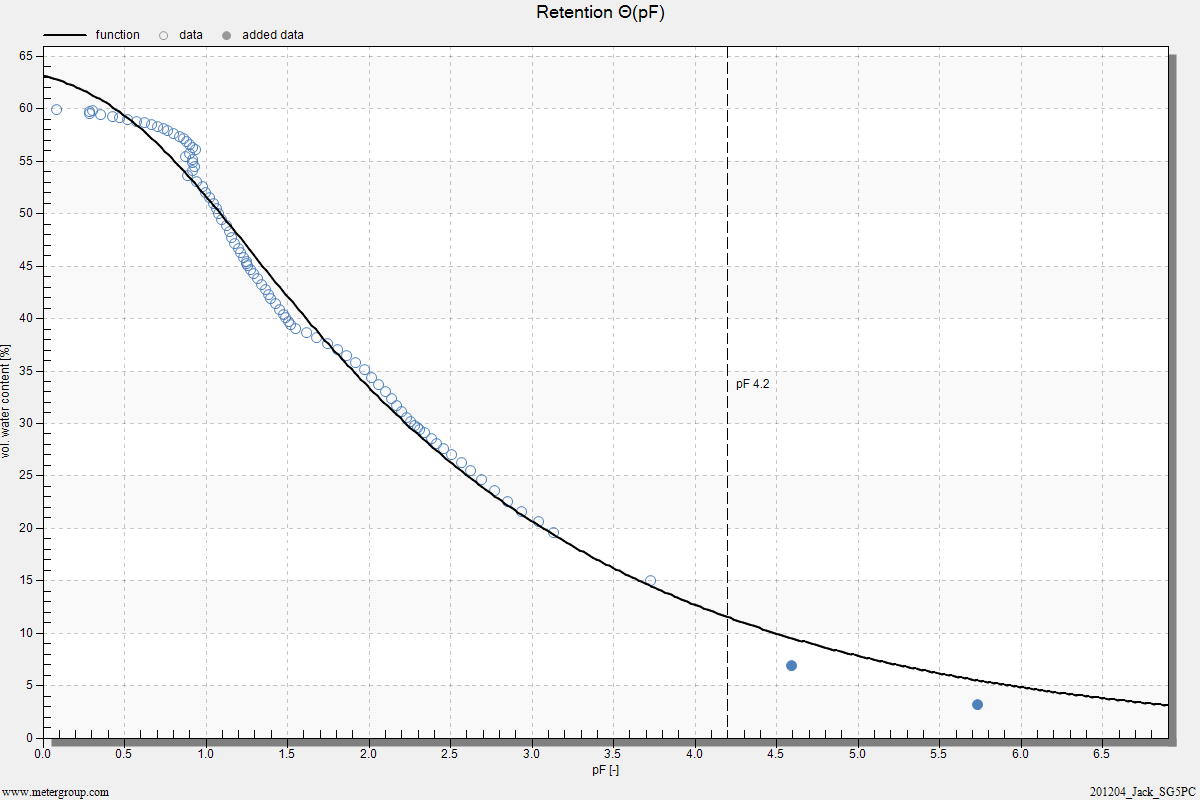

Supplement: Supplementary Data 1 — HYPROP2 and additional data on soil hydraulic properties. [file Data_Sheet_1.ZIP › SupplementaryData1/Soil Water Retention Curve.png]

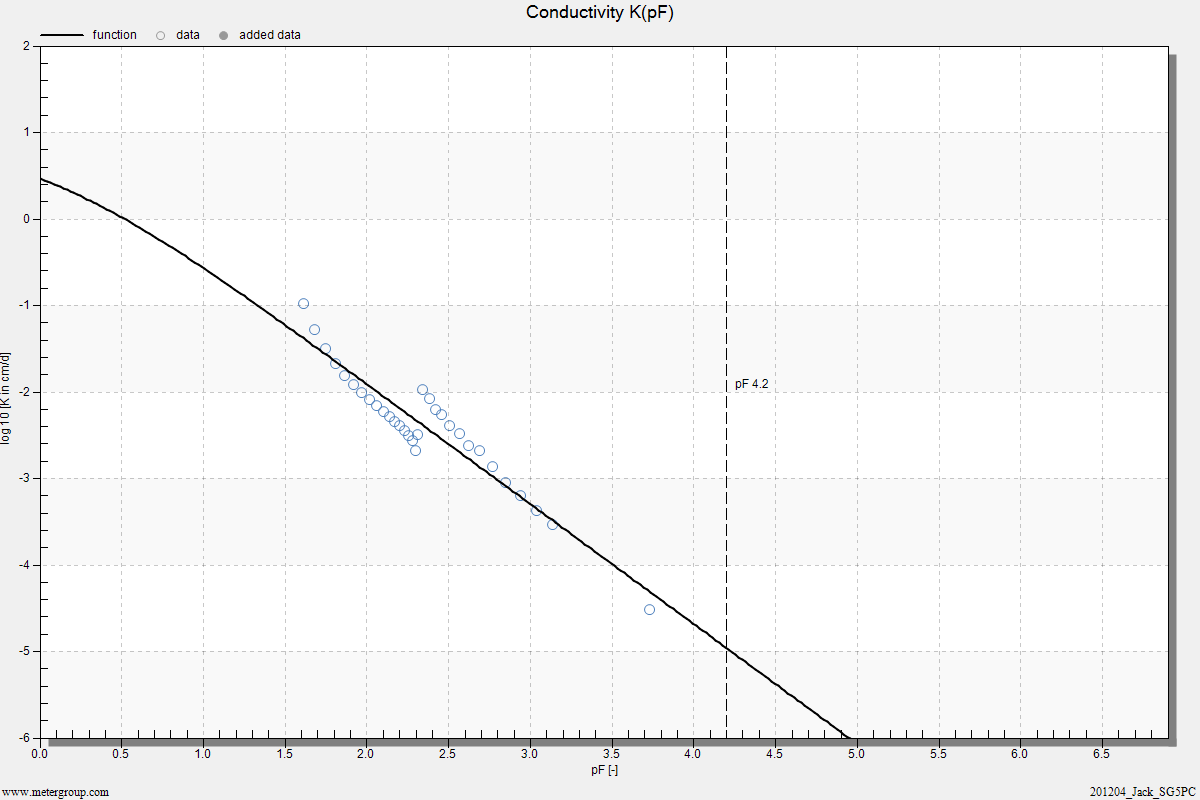

Supplement: Supplementary Data 1 — HYPROP2 and additional data on soil hydraulic properties. [file Data_Sheet_1.ZIP › SupplementaryData1/Soil Conductivity vs Potential.png]
